# Friendship Network and School Socialization Correlates of Adolescent Ethnic-Racial Identity Development

**DOI:** 10.1007/s10964-024-02052-0

**Published:** 2024-07-18

**Authors:** Olga Kornienko, Adriana J. Umaña-Taylor, Maciel M. Hernández, Thao Ha

**Affiliations:** 1 Department of Psychology, George Mason University, Fairfax, VA USA; 2 Harvard Graduate School of Education, Harvard University, Cambridge, MA USA; 3 Department of Human Ecology, University of California, Davis, CA USA; 4 Department of Psychology, Arizona State University, Tempe, AZ USA

**Keywords:** Ethnic-racial identity exploration, Ethnic-racial identity resolution, Ethnic-racial identity negative affect, Peer ethnic-racial socialization, School ethnic-racial socialization, Friend networks

## Abstract

Ethnic-racial identity (ERI) development is consequential for youth adjustment and includes exploration, resolution, and affect about the meaning of one’s ethnic-racial group membership. Little is known about how identity-relevant experiences, such as ethnic-racial socialization and discrimination in peer relationships and school contexts, catalyze adolescent ERI development. The present study examines how identity-relevant experiences in friend and school contexts (i.e., proportion of same-ethnoracial friends, cultural socialization among friends, friends’ ERI dimensions, friends’ experiences of ethnoracial discrimination, and school promotion of cultural competence and critical consciousness) are associated with ERI development. A multivariate path model with a sample from four southwestern U.S. schools (*N* = 717; 50.5% girls; *M*_age_ = 13.76; 32% Latinx, 31.5% Multiethnic, 25.7% White, 11% other) was used to test these associations. Findings showed that friend and school predictors of ERI did not differ between early and middle adolescents, but significant differences and similarities emerged in some of these associations between ethnoracially minoritized and White youth. Specifically, friend cultural socialization was positively associated with ERI exploration for ethnoracially minoritized youth only, whereas school critical consciousness socialization was positively linked with ERI exploration only for White youth. Friend cultural socialization and friend network’s levels of ERI resolution were positively associated with ERI resolution across both ethnoracial groups. These friend and school socialization associations were documented above and beyond significant contributions of personal ethnoracial discrimination to ERI exploration and negative affect for both ethnoracially minoritized and White youth. These findings expand our understanding of how friend and school socialization mechanisms are associated with adolescent ERI development, which is vital to advancing developmental theory and fostering developmental competences for youth to navigate their multicultural yet socially stratified and inequitable world.

## Introduction

The increasing ethnoracial diversity of youth in the U.S. and globally (U.S. Census Bureau, 2021; Pew Research Center, 2019) underscores the importance of understanding and fostering adolescent ethnic-racial identity (ERI) development. ERI development is a universal task, but it is especially crucial for youth of color who are at risk of experiencing ethnoracial discrimination because these experiences disrupt their positive development (Umaña-Taylor & Rivas-Drake, 2021). Robust evidence from meta-analytic and systematic reviews reveals that ERI promotes adolescent psychological and academic adjustment (Miller-Coto & Byrnes, 2016; Yip et al., 2019b). Furthermore, cultural-ecological models of minoritized youth development highlight family, peers, and schools as vital agents of ethnoracial socialization that inform ERI development (García Coll et al., 1996; Hughes et al., 2016), although most research focused on the role of parents (Umaña-Taylor & Hill, 2020). Recently, more attention has been given to ERI development in the context of peers and friends (Kornienko et al., 2015) and schools (Saleem & Byrd, 2021). Peer relationships and schools catalyze ERI development by providing identity-relevant experiences, such as ethnoracial socialization and discrimination (Williams et al., 2020). Studies show that adolescents’ ERI development is supported by same-ethnoracial friends (Derlan & Umaña-Taylor, 2015), selection and socialization in friendship networks (Rivas-Drake et al., 2017), and cultural socialization in schools (Byrd & Legette, 2022). However, studies that jointly examine these mechanisms within peer and school contexts are rare, despite theoretical calls to increase attention to the role of peers, schools, and other social settings in ERI development (e.g., Seaton et al., 2017; Syed et al., 2018). To understand and foster ERI development, the present study examines the role of various socialization agents and settings by addressing the question: How do friends and schools contribute to ERI development? Specifically, this study examined how identity-relevant experiences in peer and school contexts (i.e., same-ethnoracial friends, cultural socialization among friends, friends’ ERI dimensions, friends’ experiences of ethnoracial discrimination, and school promotion of cultural competence and critical consciousness) were simultaneously associated with adolescents’ ERI exploration, resolution, and negative affect.

### ERI Development in Adolescence

Identity formation is a normative developmental task across the lifespan, and it is particularly salient during adolescence (Erikson, 1968). Although identity is multi-dimensional, in contemporary societies where socially constructed ethnoracial hierarchies result in inequitable systems of power, oppression, and access to resources (Rogers et al., 2020; Williams et al., 2020), ERI is a universally relevant construct (Umaña-Taylor, 2023). ERI includes the developmental processes of learning about one’s ethnoracial group membership (*ERI exploration)* and developing a sense of clarity regarding the personal meaning of one’s ethnoracial group membership for one’s sense of self (*ERI resolution*; Umaña-Taylor et al., 2014). In addition to these *process* dimensions of ERI, an important *content* dimension is *ERI affect*, or the positive or negative feelings individuals hold towards their ethnic-racial group (Umaña-Taylor et al., 2014). Social identity theory suggests that identity development is highly context-dependent, and individuals develop an understanding of the self based on their salient social group memberships, evaluations of those social groups, and perceptions of how others evaluate their social groups (Tajfel & Turner, 1986). Individuals strive for comparatively positive evaluations, as these contribute positively to one’s sense of worth and are believed to promote a positive self-concept. In contrast, comparatively negative evaluations can lead to negative feelings about the self, and such negative affect, particularly in relation to ERI, has been positively linked with maladjustment (Umaña-Taylor, 2024).

Given that the development of the content dimension of ERI is relational and comparative, it follows that messages adolescents receive from their peers, may play a significant role in the affect they feel toward their ethnoracial group. Importantly, existing evidence has documented a robust positive association between negative racial affect and adolescents’ maladjustment (e.g., Umaña-Taylor, 2024). If peers inform each other’s negative ERI affect, peer characteristics and experiences may provide an especially fruitful intervention target to disrupt the development of adolescents’ negative ERI affect.

### Role of Multiple Characteristics of Friendship Networks in ERI Development

During adolescence, individuals spend increasingly more time with their peers and friends, whose feedback and acceptance provide a strong basis for adolescents’ developing self-concept (Brechwald & Prinstein, 2011). When it comes to ERI development, peers and friends provide each other with many opportunities to learn about themselves and others through various ethnic, cultural, and racialized socialization experiences (Williams et al., 2020). Research has identified multiple ways in which friends matter, including friend ethnoracial composition, frequency of cultural socialization among friends, friends’ experiences of discrimination, and friends’ personal meaning and enactments of ERI. To date, these mechanisms have only been examined in isolation and the present study provides an initial test of how multiple friend network characteristics simultaneously inform ERI.

#### The proportion of same-ethnoracial friends

When considering the role of the ethnoracial composition of friends for ERI development, distinct mechanisms have been explored in the literature (Syed et al., 2018). On one hand, developmental models of ERI suggest that a higher proportion of same-ethnoracial friends catalyzes ERI development because in the context of these peer relationships youth engage with identity-relevant resources and community events that inform ERI exploration, resolution, and positive affect^1^ (Syed et al., 2018). In support of this, evidence indicates that being embedded in peer contexts with a higher proportion of same-ethnoracial peers has been linked with higher levels of ERI positive affect among Black youth (Derlan & Umaña-Taylor, 2015) and higher ethnic identity exploration, positive affect, and belonging among ethnically diverse youth (Kiang et al., 2007; 2010; Phinney et al., 2001). On the other hand, self-categorization theory would suggest that a higher proportion of cross-ethnoracial friends fosters ERI development because a low representation of one’s ethnic or racial group among peers can increase the salience of one’s own group membership (Turner et al., 1987), which in turn can promote greater ERI exploration and ultimately ERI resolution (Syed et al., 2018). In line with this notion, studies have found that being embedded in cross-ethnoracial peer relationships and friendships was positively associated with adolescents’ concurrent levels of ERI centrality (Kiang et al., 2010) and prospective increases in ERI exploration (Rivas-Drake et al., 2017). Given these competing theoretical notions and mixed empirical findings, this study explored whether the proportion of same-ethnoracial friends was associated with ERI exploration, resolution, and negative affect in a positive or negative manner.

#### Friend cultural socialization

A smaller body of work has examined the frequency and nature of messages through which friends transmit knowledge, behaviors, and attitudes about what it means to be a part of one’s culture (Hughes et al., 2011). This scholarship has shown that adolescents develop their ERI together with their friends by participating in activities where they learn and use heritage language, engage in cultural activities and traditions, and celebrate one’s culture (Sladek et al., 2022). Further, a daily diary study showed that positive ethnoracial peer processes, which were operationalized as cultural socialization and support against discrimination, were positively associated with next day increases in ERI positive affect (Wang, 2021). Beyond these within-person dynamics, between-person results showed that peer cultural socialization was positively related to ERI centrality, positive affect, and public regard (Wang, 2021). Given this emergent evidence, it was hypothesized that friend cultural socialization would be positively related to the process aspects of ERI (i.e., exploration and resolution). In the absence of existing evidence linking friend cultural socialization explicitly to ERI negative affect, the present study explored this association without a directional hypothesis.

#### Friends’ ERI dimensions

The notion that friends are powerful agents of ethnoracial socialization catalyzing ERI development has been long-theorized (Hughes et al., 2011; Spencer, 2006) and is now receiving empirical support. For instance, in conversations with friends, adolescents explore and make meaning of their ERI by talking about topics like discrimination, positive connections to their own culture, awareness of cultural differences, underrepresentation in professional and academic settings, and awareness of white^2^ privilege and colorblindness (Moffit & Syed, 2021). In another study, the frequency of identity-related conversations with friends, but not the relationship quality, was associated with producing similarity among youth of color on ERI exploration and resolution (Syed & Juan, 2012). Furthermore, social network-informed studies have documented that ERI development informs how adolescents select their friendship networks and how they become influenced by their friends’ levels of ERI process and content. In one study, adolescents from the U.S. were found to select their friendship networks and influence each other to become similar regarding ERI exploration and resolution (Rivas-Drake et al., 2017). In another study, ethno-racially diverse youth from the U.S. were found to select friends who were similar in terms of ERI centrality, and, over time, youth were found to socialize each other to become similar in terms of ERI centrality, positive affect, and public regard (Santos et al., 2017). Finally, in a sample of native and immigrant-origin youth from Germany, peer influence on ERI attachment and positive affect occurred among same-group friends and not cross-group friends (Jugert et al., 2020). Together, these studies suggest that friend network’s levels of ERI dimensions are positively associated with those of a focal individual. Accordingly, it was expected that friends’ levels of ERI exploration, resolution, and negative affect would be positively associated with focal adolescents’ ERI levels on the respective ERI dimension.

#### Friends’ experiences of discrimination

Although no published research to-date has explicitly tested the impact of vicarious discrimination via friends on adolescents’ ERI development, theory and empirical evidence point to the possibility of such linkages. For instance, developmental theory suggests that personal experiences of racial discrimination serve as catalyzing experiences for ERI development (Williams et al., 2020) and youth of color draw on their peer relationships to process ethnic-racial identity- and discrimination-related experiences (Spencer, 2006). Further, evidence indicates that having clarity and resolution about one’s ERI protects against negative effects of online vicarious discrimination on mental health and self-esteem (Umaña-Taylor, Tynes, et al., 2015). Thus, this study explored whether vicarious discrimination reported by friends would be positively associated with focal adolescent’s ERI exploration and resolution. Further, this study did not pose any research questions about the associations between vicarious discrimination and ERI negative affect because ERI negative affect is concerned with consequences of viewing one’s own ethnoracial group in negative light for one’s self-concept (Umaña-Taylor, 2024), which implies that only experiences of discrimination from same-ethnoracial group friends could matter, and this study focused on both same- and cross-group friends’ experiences of discrimination.

### School Ethnoracial Socialization and ERI Development

Youth are developing their ERI in the context of their interactions with teachers, staff, and peers in school, and the policies and practices therein communicate messages about ethnicity and race (Saleem & Byrd, 2021; Williams et al., 2020). Educators are salient yet understudied agents of ethnoracial socialization for racially marginalized youth and White youth (Heberle et al., 2020; Golden & Byrd, 2022; Saleem & Byrd, 2021; Sladek et al., 2022). *Promotion of cultural competence* (i.e., learning about other ethnoracial groups and group histories) and *critical consciousness socialization* (i.e., learning about power, privilege, and systems of oppression; Saleem & Byrd, 2021) are two school socialization mechanisms theorized to promote ERI by providing youth with opportunities for reflection and meaning making of their cultural and racialized experiences (Diemer et al., 2016; Mathews et al., 2020; Sladek et al., 2022). In promoting cultural competence and engaging in critical consciousness socialization, schools signal that learning about one’s own ERI and its role in disrupting (or perpetuating) social inequity holds value.

#### Promotion of cultural competence

Evidence indicates that African American and Latinx youth in the U.S. report encountering high frequency but somewhat limited content (i.e., specific historical events and figures) of messages focused on promoting cultural competence in schools (Byrd & Hope, 2020; Sladek et al., 2022). Emerging evidence has linked school and teacher promotion of cultural competence to U.S. youths’ exploration of and commitment to their ethnoracial identities (Brown & Chu, 2012; Camacho et al., 2018; Schachner et al., 2016; Smith et al., 2020). In Germany, native-born and immigrant youth who perceived more cultural pluralism socialization in school also reported greater levels of ERI exploration and commitment and better psychosocial adjustment (Schachner et al., 2016). However, some studies testing the association between the promotion of cultural competence and ERI report null associations (Byrd & Legette, 2022; Chang & Le, 2010) or patterns conditional on the time of assessment (i.e., from spring to fall assessment, but not from fall to the next spring assessment; Camacho et al., 2018). The mixed evidence for the association between the promotion of cultural competence and ERI warrants further study. Nevertheless, given the importance of educators as promoters of cultural competence (Saleem & Byrd, 2021), significant and positive associations between school promotion of cultural competence were expected for ERI exploration and resolution. In the absence of prior evidence regarding ERI affect, the present examination of the links between school promotion of cultural competence and ERI negative affect was exploratory.

#### Critical consciousness socialization

Critical consciousness and ERI development are theorized to inform each other and reciprocally evolve (Mathews et al., 2020). Specifically, Mathews et al. postulate that (a) race-based experiences – including socialization to promote critical consciousness or preparation for discrimination—encourage ERI exploration and (b) that awareness of one’s politicized history – which critical consciousness socialization might foster – predicts ERI resolution. Mathews et al. also propose that ERI process dimensions (i.e., exploration and resolution), rather than ERI content (e.g., negative ERI affect), are most closely linked to the critical reflection aspect of critical consciousness (i.e., awareness of social injustice), suggesting that critical consciousness socialization might predict more ERI exploration and resolution, but not ERI negative affect. One study revealed that school critical socialization was linked to youth awareness of racism and promoted ERI exploration and commitment among diverse youth (Byrd, 2016). Other work reveals significant and positive bivariate associations between school critical consciousness socialization and ERI exploration and resolution among adolescents, which become null once cultural, mainstream, and color-blind aspects of school ethnoracial socialization are accounted for (Byrd & Legette, 2022). Although evidence is still accumulating to resolve these mixed findings, theoretical accounts emphasize the developmental importance of and the transactional associations between critical consciousness and ERI development (Mathews et al., 2020), which need to be examined in the context of socialization in schools (Saleem & Byrd, 2021). Given this prior work, positive associations were hypothesized between school critical consciousness socialization and adolescents’ ERI exploration and resolution. In addition, the link between critical consciousness socialization and adolescents’ ERI negative affect was explored.

In summary, developmental theory and evidence reviewed thus far highlight the potential role of multiple friendship and school factors to be associated with adolescent ERI exploration, resolution, and negative affect. To provide a robust test of these associations, it is incumbent to document them above and beyond contributions of personal experiences of ethnoracial discrimination for ERI. Developmental theory suggests that adolescent personal experiences of ethnoracial discrimination catalyze ERI development because discrimination may prompt youth to seek information about their ethnoracial group leading to ERI exploration (Williams et al., 2020). Indeed, evidence indicates that experiencing ethnoracial discrimination was positively associated with ERI exploration in samples of Black and Latino middle and late adolescents (Brittian et al., 2015; Pahl & Way, 2006). Therefore, given the powerful impact of personal experiences of ethnoracial discrimination on ERI development, the present study seeks to provide a robust examination of friend and school influences on ERI development, while accounting for adolescent own experiences of ethnoracial discrimination.

### Developmental Period and Ethnoracial Positionality as Moderators

#### Developmental period

Developmental and contextual factors shape how youth explore, resolve, and feel about the meaning of their ethnic heritage and racial background for their sense of self (Williams et al., 2020). Prior research has identified general patterns of age-graded change in ERI developmental processes such that youth’s ERI exploration and resolution significantly increase from early to late adolescence due to enhanced cognitive and meaning-making abilities (Umaña-Taylor, 2018). Developmental changes in ERI affect throughout adolescence have also emerged, yet appear to be relatively more context-dependent, such that change is a function not only of age-graded development but also of whether youth are in contexts in which their ethnoracial group is a numerical majority or minority (Umaña-Taylor, 2016). Consistent with self-categorization (Turner et al., 1987) and social identity (Tajfel & Turner, 1986) theories, the increased salience and potential sense of threat that comes with being a numerical minority can prompt changes in ERI affect. Importantly, these age- and context-dependent trends that inform ERI development during adolescence are coupled with ongoing identity-relevant inputs such as ethnic-racial socialization from peers, friends, and educators during adolescence. With respect to developmental period, it is possible that the influence of identity-relevant inputs on adolescents’ ERI development may be relatively more impactful as adolescents age. Given age-contingent increases in social and cognitive maturity from early to middle adolescence, this study hypothesized that the associations between friend and school socializing factors and ERI exploration, resolution, and negative affect would be stronger among older than younger adolescents (i.e., 9th vs. 6th graders).

#### Ethnoracial minoritized/majority status

Consistent with notions introduced above, the lifespan model of ERI development underscores that indicators of social position (e.g., belonging to ethnoracial minoritized versus majority groups) can inform variability in how youth develop their ERI (Williams et al., 2020). As adolescents develop an understanding of the meaning of ethnicity and race for their self-concept, these processes are inextricably linked to their understanding of the position that their social group occupies in the larger society (Umaña-Taylor et al., 2014). Further, in contemporary societies where socially constructed ethnoracial hierarchies result in inequitable systems of power and access to resources, there likely is variability in the relational processes through which ERI development is constructed for ethnoracial minoritized versus White youth (Rogers et al., 2020).

Indeed, White youth are expected to differ from ethnoracial minoritized youth in their understanding and meaning-making of their ERI (e.g., focus on heritage, or national identities rather than race) given that society’s emphasis on white normativity perpetuates a perspective that White youth’s racial experiences are normative (i.e., the objective standard) and thus not in need of interrogation or exploration (Moffit & Rogers, 2022). Relatedly, race-evasive socialization practices are common among White families and can further perpetuate this message of whiteness as normative (Satterthwaite-Freiman & Umaña-Taylor, 2023). Race-evasive parental socialization practices lead to diminished opportunities for White youth to explore and resolve the meaning of their racial group membership for their self-concept, and this is evidenced in existing findings in which White youth tend to report lower ERI exploration and resolution compared to ethnoracial minoritized youth (Moffit et al., 2021; Satterthwaite-Freiman & Umaña-Taylor, 2023). Related to the current study, this pattern of the absence of ethnoracial socialization among White families may result in ethnoracial socialization messages from friends and educators being particularly influential for White youth’s ERI (e.g., Loyd & Gaither, 2018).

Whereas the majority of research has focused on the development of ERI among ethnoracial minoritized youth, there has been a recent growth in research on ERI development among White youth (e.g., Rogers et al., 2020; Satterthwaite-Freiman & Umaña-Taylor, 2023). This attention is warranted because White adolescents’ ERI development has implications not only for their self-concept and adjustment but also for the development of their anti-racist attitudes and behaviors that are essential to challenge and rectify social and racial inequities (Mathews et al., 2020; Rogers et al., 2020). As such, the current study explored differences between ethnoracially minoritized and White youth in the strength and direction of relational processes through which friends and school socialization processes were associated with ERI exploration, resolution, and affect.

## The Present Study

Informed by cultural-ecological models of minoritized youth development and the lifespan model of ERI development, this study examined how friend and school ethnoracial socialization was associated with ERI development, and whether developmental period and ethnoracial minoritized/majority status moderated these associations. These theories emphasize that peers and schools are proximal, salient, and, thus, equally important socialization agents for ERI development. Accordingly, the investigation of multiple peer and school socialization mechanisms was exploratory and did not presuppose a differential strength in the impact of peer versus school socialization. Based on prior work, it was hypothesized that friend’s cultural socialization and friends’ ERI dimensions would each be positively associated with adolescents’ ERI exploration, resolution, and negative affect. It was also expected that friends’ experiences with ethnoracial discrimination (i.e., vicarious discrimination) would each be positively associated with adolescents’ ERI exploration and resolution. Furthermore, school promotion of cultural competence and critical consciousness socialization were each hypothesized to be positively related to ERI exploration and resolution. Finally, to provide a robust test of friend and school socialization influences on ERI development, the present study examined these predictors while accounting for associations between adolescents’ personal experiences of ethnoracial discrimination and their ERI. Several exploratory research questions were examined, given limited prior research. These included: the association between school promotion of cultural competence and critical consciousness socialization with ERI negative affect, and the association between proportion of same-ethnoracial friends and all three ERI dimensions. Next, developmental period was tested as a moderator of all associations, and it was hypothesized that all indicators of peer and school socialization would be more strongly associated with ERI development for middle vs. early adolescents (9^th^ vs. 6^th^ grade). Finally, ethnoracial minoritized/majority status was also tested as a moderator of all associations, though this was an exploratory research question due to limited prior research.

## Methods

### Participants

The sample included sixth and ninth-grade students from four public schools in the southwestern U.S. (*n* = 717; 50.5% girls; M age = 13.76; 31.8% Hispanic/Latinx, 31.5% Multiethnic, 25.7% White, 7.3% Black or African American, 1.4% Asian American or Pacific Islander (AAPI), 1.4% American Indian or Alaska Native (AI/AN), and 1% Arab, Middle Eastern, or North African (AMENA)). A majority of multiethnic youth identified Hispanic/Latinx (67%) as one of their ethnic group memberships. In terms of generational status, most participants (67.7%) were 3rd generation (both parents and child born in the U.S.), 16.5% were 2.5 generation with one parent born abroad and another parent and child born in the U.S., 10.6% were 2nd generation with both parents born abroad and child born in the U.S., and 3.6% were 1st generation with both parents and child born abroad. Regarding perceived socio-economic status, 2% of youth reported that they did not have enough money to get by, 30.3% reported that they had just enough, 35.5% stated that they only have to worry about money for fun and extras, and 22% stated that they never had to worry about money.

The ethnic-racial composition for participants from each school, which generally mirrored the broader school ethnic-racial demographics, was as follows : School 1 (6th grade): 45% Hispanic/ Latinx, 27% Multiethnic, 15% White, 7% Black or African American, 2% AAPI, 2% AI/AN, and 1% AMENA; School 2 (6th grade): 48% Hispanic/Latinx, 33% Multiethnic, 3% White, and 16% Black or African American, 0% AAPI, 0% AI/AN, and 0% AMENA; School 3 (9th grade): 11% Hispanic/Latinx, 30% Multiethnic, 53% White, 2% Black or African American, 1% AAPI, 1% AI/AN, and 2% AMENA; and School 4 (9th grade): 28% Hispanic/Latinx, 35% Multiethnic, 26% White, 8% Black or African American, 1% AAPI, 1% AI/AN, and 1% AMENA. Schools 1 (*n* = 222) and 2 (*n* = 58) were middle schools, and schools 3 (*n* = 132) and 4 (*n* = 305) were high schools. Considering perceived socio-economic status in each school, descriptive analyses revealed that in School 1, 1.09% of youth reported that they did not have enough money to get by, 39.67.3% reported that they had just enough, 28.26% stated that they only have to worry about money for fun and extras, and 30.98% stated that they never had to worry about money; School 2, 3.70% of youth reported that they did not have enough money to get by, 50.00% reported that they had just enough, 24.07% stated that they only have to worry about money for fun and extras, and 22.22% stated that they never had to worry about money; School 3, 1.59% of youth reported that they did not have enough money to get by, 15.87% reported that they had just enough, 53.97% stated that they only have to worry about money for fun and extras, and 28.57% stated that they never had to worry about money; in School 4, 3.19% of youth reported that they did not have enough money to get by, 37.94% reported that they had just enough, 40.43% stated that they only have to worry about money for fun and extras, and 18.44% stated that they never had to worry about money.

### Procedure

Students from two public high schools and two public middle schools in a metropolitan city in the Southwestern U.S. were sent home parental consent letters in both English and Spanish. Students received $10 for returning their signed parental consent forms, regardless of their study participation decision. Teachers were provided with $50 and two movie tickets in appreciation for their efforts in reminding students to return consent forms. Participating students with signed parental consent forms provided assent prior to completing their surveys. Across the four schools, participation rates ranged from 70.66% to 81.46%. These study procedures were approved by Arizona Stat University’s institutional review board. Data collection took place in December 2019 and early January 2020 (prior to the COVID pandemic). Participants completed self-report questionnaires in English during their regular school hours over two class periods (approximately 90 minutes total). School staff and research project assistants were available to answer questions during data collection.

### Measures

#### ERI development

The Ethnic Identity Scale (EIS-B; Douglass & Umaña-Taylor, 2015) assessed three different domains of ERI: exploration (mean across 3 items; Cronbach’s α = 0.77; e.g., “I have attended events that have helped me learn more about my ethnicity”), resolution (mean across 3 items; Cronbach’s α = 0.88; e.g., “I know what my ethnicity means to me”), and negative affect (mean across 3 items; Cronbach’s α = 0.86; e.g., “I dislike my ethnicity”). Participants were asked to think about the ethnoracial group they felt most a part of when answering the questions. Responses were rated on a 1 to 4 scale (i.e., 1 (does not describe me at all), 2 (describes me a little), 3 (describes me well), and 4 (describes me very well)) and subscales were coded so that higher scores on each subscale indicated greater ERI *exploration, resolution, and negative affect*, respectively. The scale’s three-factor structure has been supported with Latino adolescents, and strong measurement invariance has been found across ethnoracial groups in a sample of high school students (Sladek et al., 2021).

#### Ethnic and racial discrimination

The Everyday Discrimination Scale (Sternthall et al., 2011) was used to measure perceived experiences of and reasons for discrimination. The 5-item scale asks participants to indicate the frequency that they have experienced unfair treatment in five different interpersonal experiences (e.g., “You receive poorer service than other people at restaurants or stores”). The frequency responses were rated on a scale from 0 (never) to 5 (almost every day), and the *frequency of discrimination composite* was computed by taking a mean of the five items assessing frequency of everyday discrimination (Cronbach’s α = 0.81). Participants were asked to attribute the reason for those five experiences (e.g., race, ethnicity, national origin, religion, gender, income, education). To score this scale, if a participant selected race (27.9% of respondents endorsed), ancestry or national origin (8.2%), or religion (9.9%) as any of the options, they were given a binary score of 1 to indicate that they encountered any ethnic or racial discrimination. Given that race, ethnicity, immigrant-origin, and religion represent important, intertwined, and underresearched markers of social position upon which social stratification operates in the U.S. (Kiang & Supple, 2016), these factors were included into the current operationalization of ethnoracial discrimination. In total, 34.3% of youth endorsed at least one of the three reasons (race, ancestry or national origin, and religion). If they did not select race, ancestry/national origin, or religion as reasons for discrimination, they were given a score of 0 to indicate that they did not experience *ethnoracial discrimination*. To compute the frequency of ethnoracial discrimination composite, the *frequency of discrimination composite* was multiplied with the binary score for *ethnoracial discrimination*. This approach has been used in the prior work (Shariff-Marco et al., 2011); a higher score on this composite represents a higher frequency of ethnoracial discrimination, and a score of zero represents no ethnoracial discrimination.

#### Friend cultural socialization

Participants were asked how often, in the past six months, their friends engaged in cultural socialization by discussing the focal adolescent’s ethnoracial and cultural heritage. Youth responded to two questions about cultural socialization and pluralism (Nelson et al., 2018). The two items assessed overt ethnic-racial socialization about one’s *own group’s* ethnoracial and cultural heritage (i.e., “Peers encouraged you to read books about your own racial/ethnic group?;” “Peers talked to you about important people or events in the history of your own racial/ethnic groups?”). Participants used a four-point Likert-type scale ranging from 0 (never) to 4 (very often). A mean composite of the two-item friend cultural socialization scale (Cronbach’s α = 0.74) was computed such that higher scores indicated greater frequency of friend cultural socialization.

#### Friend network composites

Participants nominated up to 10 friends from their grade by selecting them from a grade roster list that contained the names of students who were participating in the study. Friends were defined as individuals the participants spent a lot of time with and whom they could count on. Outgoing friendship nominations were used to compute three distinct friend composite variables. First, *the proportion of same-ethnoracial friends* (i.e., using friends’ self-reported ethnicity/race) was computed. Then, *the sum of frequency of ethnoracial discrimination that was reported across one’s friends* (i.e., sum level of frequency of ethnoracial discrimination that was reported by all friends identified through outgoing friendship nominations) was computed and, finally, *the sum of ERI exploration, resolution, and negative affect that was reported across one’s friends* (i.e., sum level of each ERI dimension that was reported by all friends who were identified through outgoing friendship nominations). Outgoing friendship nominations are typically used in social networks research to depict friendships from the perspective of a focal individual on their social relationships, assuming that youth-reported friends are valid sources of socialization (Kornienko, 2024). Using outgoing nominations, as compared to reciprocated nominations, represents the focal adolescent’s perspective on their peer exposure and influence that is relevant to consider in this study of friend contributions to ethnoracial socialization. Finally, a sum composite of ERI and discrimination across all friends was chosen to parametrize the friend compositional effects consistent with *a total exposure to friends’ cultural and racial socialization*, rather than a mean composite that would represent *normative influence or convergence across friends* and could inadvertently miss the cumulative nature of friend socialization or discrimination as related to ERI (see Veenstra et al., 2023, for more details on network effects).

#### School ethnoracial socialization

Youth rated the extent to which their school engaged in teaching about the histories and traditions of diverse ethnoracial and cultural groups using the *promotion of cultural competence subscale* of the School Climate for Diversity Secondary Scale (Byrd, 2017). Youth responded to 5 items (Cronbach’s α = 0.92; e.g., “Your classes teach you about diverse cultures and traditions,” “You have the chance to learn about the cultures of others”) based on their experiences in the past six months, on a scale from 1 (not true at all) to 5 (completely true). Youth also rated the extent to which their school promoted critical consciousness using the *critical consciousness socialization subscale* of the School Climate for Diversity Secondary Scale (Byrd, 2017). Youth responded to 4 items (Cronbach’s α = 0.87; e.g., “Your teachers encourage awareness of social issues affecting your culture,” “You have opportunities to learn about social justice”) based on their experiences in the past six months, on a scale from 1 (not true at all) to 5 (completely true). For both subscales, higher scores indicated greater levels of school ethnoracial socialization.

#### Social position indicators

This study included three social position indicators. Gender and ethnoracial background were tested as moderators and, due to sample size, were coded as binary variables as follows: gender (i.e., 1 = girl; 0 = boy [other gender cases were coded as missing]) and ethnoracial background (i.e., Latinx, White, Multiracial, and Other Race, 1 = yes, 0 = no). Immigrant generational status was included as a control and coded as: 1 = 1st generation, child and parents born abroad; 2 = 2nd generation, child born in the U.S. and two parents born abroad; 3 = 2.5 generation, one parent foreign-born and child and the other parent are U.S.-born; 4 = 3rd generation with U.S.-born child and parents.

### Analytic Strategy

Descriptive statistics and bivariate correlations among study variables were examined prior to multivariate analyses. To test the study hypotheses, this study utilized multivariate path analysis conducted in *R* package *lavaan* (v. 0.6.16; Rosseel, 2012–2024). The adequacy of model fit was based on the consideration of multiple fit indices, using suggested cut-offs for adequate model fit (Hu & Bentler, 1999): Comparative Fit Index (CFI) > 0.90, Root Mean Square Error of Approximation (RMSEA) < 0.08, and Standardized Root Mean Square Residual (SRMR) < 0.08. Missing data across focal variables ranged between 0.06% and 9.4% and handled with Full Information Maximum Likelihood (FIML) estimation. Robust standard errors were estimated using a sandwich estimator to account for potential non-normal and non-independent data.

First, a baseline model was fit with ERI exploration, resolution, and negative affect as predicted by friendship network characteristics, school ethnoracial socialization, individual-level frequency of experiences of ethnoracial discrimination, and several dummy-coded covariates for ethnic-racial background, gender, and generational status. Because own experiences of discrimination are theorized to catalyze ERI development (Williams et al., 2020), frequency of own experiences of ethnoracial discrimination was included as a control variable in the models to provide a robust test of the associations between friend and school predictors of ERI (i.e., above and beyond contributions of own ethnoracial discrimination). Recognizing significant variability in ERI developmental processes by gender (e.g., Umaña-Taylor & Guimond, 2010) and immigrant generational status (Umaña-Taylor et al., 2013) in prior work, these sociodemographic factors were included as controls in all analyses. Then, a multigroup model was tested to examine moderation by developmental period (6th vs. 9th grade). Because the multi-group model by developmental period revealed that no paths significantly differed by age group, a new multigroup model (which included developmental period as a control) was estimated to test moderation by ethnoracial minoritized vs. White group membership. Because developmental period (i.e., 6th grade vs. 9th grade) and ethno-racial composition of a school setting (i.e., multi-ethnic vs. White-majority) are both theorized to affect ERI development (Williams et al., 2020), this study included a control for school site that was coded as follows (1) multi-ethnic 6^th^ grade (schools 1 and 2), (2) multi-ethnic 9^th^ grade (school 4), and (3) predominantly White 9th grade (school 3). School 3 was the reference group in all analyses, given it was the only school that had a majority white student body. This coding of school sites allowed simultaneously accounting for developmental period and school’s ethnoracial composition.

**Fig. 1:**
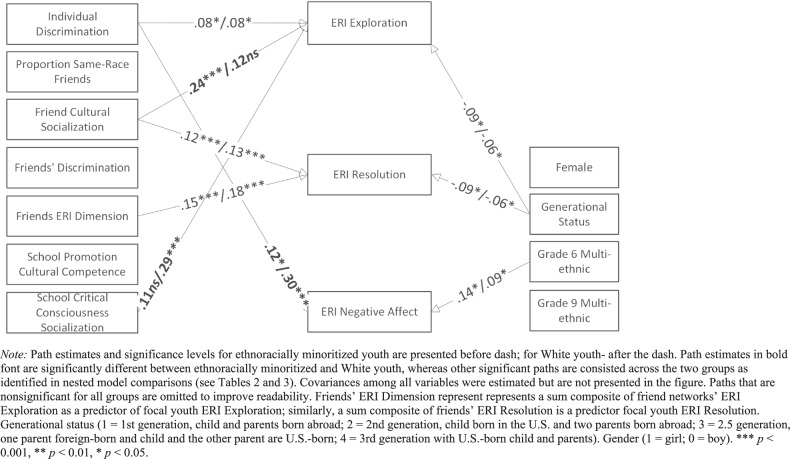
Significant standardized estimates for final multivariate path model across ethnoracially minoritized and white youth

A multi-group modeling approach was used to test whether the regression paths in this model differed between ethnoracial minoritized vs. White youth. In the first multigroup model, all paths were freely estimated (unconstrained model) across the two groups. This model was compared to a fully constrained model using a χ^2^ difference test. A nonsignificant χ^2^ difference would indicate that the models could be constrained to be equal across groups, suggesting that there were no significant differences between the two groups. A significant change in chi-square would indicate that the multigroup model could not be constrained to be equal across groups (Kline, 2023). To identify which paths differed significantly by groups, each estimated path was sequentially constrained (i.e., one path at a time), comparing the more constrained model to the less constrained model using a chi-square difference test; a significant difference in χ^2^ for a newly constrained path would indicate that, for that new constraint, estimates differed for ethnoracial minoritized vs. White youth (i.e., fail; see Table 2). One-by-one, each estimated path was constrained (or left free to vary) and this process led to the final partially constrained model (see Table 3, Fig. 1).

## Results

### Descriptive Analyses

Correlations and descriptive statistics for the key variables are presented in Table 1. To provide insights into friendship network structure and composition for the current sample, descriptive data are presented on how the number of friends and the proportion of same-group friends compare between participants who are members of different ethnoracial groups (White, Latinx, Multiracial, and other race group). Results from an ANOVA model indicated that there were no significant ethnoracial differences in the number of friends that participants reported in their grade-based network [*F*(3, 73) = 3.25, *p* = 0.807]. A second ANOVA indicated that there was a significant difference in the proportion of same-group friends within one’s grade across youth from the four ethnoracial groups [*F*(3, 663) = 28.508, *p* < 0.001]. Post hoc comparisons using the Bonferroni test indicated that the mean proportion of same-group friends did not significantly differ between White youth (*M* = 0.39, *SD* = 0.27) and Latinx youth (*M* = 0.40, *SD* = 0.25). However, both White and Latinx youths reported a significantly higher mean proportion of same-group friends than youth who identified as Multiracial (*M* = 0.30, *SD* = 0.24) or other race (*M* = 0.11, *SD* = 0.19). This pattern of differences is likely due to a higher availability of same-race friends in the grade for Latinx and White youth respectively.

**Table 1:** Descriptive statistics: means, standard deviations and bivariate correlations for ethnoracially minoritized and White youth

	1	2	3	4	5	6	7	8	9	10	11	12	*n*	Mean	*SD*	Min	Max
1. ERI Exploration	–	0.42**	0.22**	0.00	−0.06	0.14	0.42**	0.45**	0.09	0.03	−0.08	−0.19**	181	1.88	0.83	1.00	4.00
2. ERI Resolution	0.43**	–	−0.08	−0.05	−0.03	0.22**	0.30**	0.35**	0.25**	−0.04	0.02	−0.10	181	2.96	0.80	1.00	4.00
3. ERI Negative Affect	0.12**	−0.13**	–	0.31**	−0.13	−0.09	−0.12	−0.05	−0.14	0.03	−0.15	−0.02	180	1.45	0.72	1.00	4.00
4. Ethnoracial Discrim	0.09*	0.03	0.09	–	0.01	0.01	−0.15	−0.09	0.04	−0.02	−0.02	0.01	180	0.34	0.80	0.00	4.40
5. % Same-Group Fri	−0.01	0.00	0.07	−0.02	–	0.08	0.07	0.11	0.00	0.12	0.09	−0.05	169	0.39	0.27	0.00	1.00
6. Friend Cult Social	0.23**	0.19**	0.07	0.15**	−0.09	–	0.19*	0.24**	−0.04	0.01	0.38**	−0.17*	169	1.59	1.00	0.00	5.00
7. Sch Prom Cult Comp	0.16**	0.09	0.14**	−0.11*	0.07	0.03	–	0.75**	0.15*	−0.06	−0.12	−0.06	177	3.10	1.11	1.00	5.00
8. Sch Crit Cons Social	0.20**	0.11*	0.11*	−0.10*	0.05	0.12*	0.69**	–	0.23**	−0.10	−0.08	−0.02	175	2.85	1.12	1.00	5.00
9. Number Friends	0.08	0.12**	−0.00	−0.06	0.01	0.02	0.02	0.05	–	0.08	−0.07	0.14	184	5.01	2.61	0.00	10.0
10. Gender	0.04	0.04	−0.07	−0.02	0.06	0.06	−0.10*	−0.08	0.07	–	0.11	−0.02	181	0.48	0.50	0.00	1.00
11. Age (years)	−0.04	0.07	−0.13**	0.17**	−0.11*	0.44**	−0.19**	−0.18**	−0.08	0.01	–	0.05	169	14.37	1.35	10.6	19.3
12. Generational Status	−0.11*	−0.09	−0.04	0.06	−0.05	−0.11*	−0.06	−0.06	−0.03	0.00	0.16**	–	182	3.82	0.52	1.00	4.00
*n*	504	504	499	512	498	477	517	514	533	518	490	524	–	–	–	–	–
Mean	2.28	3.07	1.29	0.46	0.31	1.47	3.14	2.93	5.13	0.51	13.55	3.35	–	–	–	–	–
*SD*	0.89	0.88	0.61	0.92	0.26	1.08	0.99	1.08	2.66	0.50	1.59	0.91	–	–	–	–	–
Min	1.00	1.00	1.00	0.00	0.00	0.00	1.00	1.00	0.00	0.00	10.9	1.00	–	–	–	–	–
Max	4.00	4.00	4.00	4.20	1.00	5.00	5.00	5.00	10.0	1.00	20.	4.00	–	–	–	–	–

Descriptives for ethnoracially minoritized youth are presented below diagonal; Descriptives for White youth are presented above diagonal; ERI = Ethnic-Racial Identity; Number of friends = outgoing friend nominations from grade; Gender (1 = girl; 0 = boy). Generational status (1 = 1st generation, child and parents born abroad; 2 = 2nd generation, child born in the U.S. and two parents born abroad; 3 = 2.5 generation, one parent foreign-born and child and the other parent are U.S.-born; 4 = 3rd generation with U.S.-born child and parents)

**p < 0.01, *p < 0.05

### Testing Moderation by Developmental Period

After fitting a baseline model, analysis proceeded to test whether paths between friend and school relational predictors for each ERI dimension differed significantly between early vs. middle adolescents (6th vs. 9th grade). Comparing the unconstrained model to the fully constrained model by grade revealed a non-significant chi-square difference (Δχ^2^ (36) = 41.83, *p* = 0.23), suggesting that there was no evidence that the estimated paths differed significantly between 6th and 9th graders.

### Testing Moderation by Ethnoracial Minoritized/Majority Status

The next step was to examine whether regression paths differed significantly between ethnoracially minoritized and White youth. Comparing the fully constrained and a fully unconstrained model by ethnoracial status revealed a significant chi-square difference (Δχ^2^ (27) = 55.98, *p* < 0.001) suggesting that the friend and school socialization predictors of ERI dimensions significantly differed between ethnoracially minoritized and White youth. Given this significant chi-squared difference, a series of sequentially more constrained models were tested and chi-square difference tests were used to identify which paths needed to be freely estimated for ethnoracial minoritized and White youth (Table 2). Findings from the final model indicated that several regression paths differed significantly between ethnoracially minoritized and White youth (Table 3, see Fig. 1 for a graphic representation). The final model provided adequate fit to the data, as evidenced by multiple model fit indices: *χ*^*2*^(39) = 44.494, *p* = 0.252; CFI = 0.986; TLI = 0.971; SRMR = 0.016; RMSEA = 0.019 (90% CI: 0.00–0.045).

**Table 2:** Nested model comparisons and chi-square difference tests for multigroup equality constraints across ethnoracially minoritized and white youth

Model	Paths constrained	Compare	chi2	diff(df)	*p*	Pass/fail
1	Unconstrained model	n/a	n/a	n/a	n/a	
2	Fully constrained model	2, 1	58.61	-33.00	0.00**	Fail
	Pairwise comparisons					
3	Own discrim to ERI EXP const.	3, 1	0.34	(1)	0.56	Pass
4	Own discrim to ERI RES const.	4, 3	0.06	(1)	0.81	Pass
5	Own discrim to ERI NA const.	5, 4	5.56	(1)	0.02*	Fail
6	% same-group to ERI EXP const.	6, 5	0.71	(1)	0.40	Pass
7	% same-group to ERI RES const.	7, 6	0.30	(1)	0.58	Pass
8	% same-group to ERI NA const.	8, 7	4.07	(1)	0.04*	Fail
9	Friend cult soc to ERI EXP const.	9, 8	3.71	(1)	0.05*	Fail
10	Friend cult soc to ERI RES const.	10, 9	0.19	(1)	0.67	Pass
11	Friend cult soc to ERI NA const.	11, 10	1.52	(1)	0.22	Pass
12	Friend discrim to ERI EXP const.	12, 11	0.59	(1)	0.44	Pass
13	Friend discrim to ERI RES const.	13, 12	2.89	(1)	0.09	Pass
14	Friend discrim to ERI NA const.	14, 13	3.51	(1)	0.06	Pass
15	Friend ERI EXP to ERI EXP const.	15, 14	1.44	(1)	0.23	Pass
16	Friend ERI RES to ERI RES const.	16, 15	2.42	(1)	0.12	Pass
17	Friend ERI NA to ERI NA const.	17, 16	3.99	(1)	0.05*	Fail
18	Sch prom cult to ERI EXP const.	18, 17	0.22	(1)	0.64	Pass
19	Sch prom cult to ERI RES const.	19, 18	0.02	(1)	0.88	Pass
20	Sch prom cult to ERI NA const.	20, 19	21.21	(1)	0.00**	Fail
21	Sch CC to ERI EXP const.	21, 20	6.31	(1)	0.01*	Fail
22	Sch CC to ERI RES const.	22, 21	2.81	(1)	0.09	Pass
23	Sch CC to ERI NA const.	23, 22	2.99	(1)	0.08	Pass
24	Gender to ERI EXP const.	24, 23	0.02	(1)	0.89	Pass
25	Gender to ERI RES const.	25, 24	1.26	(1)	0.26	Pass
26	Gender to ERI NA const.	26, 25	1.86	(1)	0.17	Pass
27	Generat. status to ERI EXP const.	27, 26	3.42	(1)	0.06	Pass
28	Generat. status to ERI RES const.	28, 27	0.03	(1)	0.86	Pass
29	Generat. status to ERI NA const.	29, 28	0.25	(1)	0.62	Pass
30	Grade 6 multieth to ERI EXP const.	30, 29	0.235	(1)	0.63	Pass
31	Grade 6 multieth to ERI RES const.	31, 30	2.346	(1)	0.13	Pass
32	Grade 6 multieth to ERI NA const.	32, 31	1.371	(1)	0.24	Pass
33	Grade 9 multieth to ERI EXP const.	33, 32	0.073	(1)	0.79	Pass
34	Grade 9 multieth to ERI RES const.	34, 33	0.004	(1)	0.95	Pass
35	Grade 9 multieth to ERI NA const.	35, 34	0.854	(1)	0.36	Pass

Note: Pass/fail refers to whether the model constraints resulted in significantly worse (i.e., fail) or equivalent (i.e., pass) fit relative to the comparison model. *p < .05, **p < .01.

**Table 3:** Parameter estimates for a partially constrained final model across ethnoracially minoritized and White youth

	ERI Exploration			ERI Resolution			ERI Negative Affect		
	Est	SE	*p*	Est	SE	*p*	Est	SE	*p*
Non-white youth (*n* = 533)									
Own ER Discrim	0.08	0.04	0.03*	0.00	0.04	0.94	0.12^c^	0.05	0.02*
% Same-Group Friends	−0.04	0.04	0.27	−0.01	0.04	0.79	0.06^d^	0.05	0.18
Friend Cultural Socialization	0.24^a^	0.05	0.00***	0.12	0.04	0.00***	0.12	0.06	0.07
Friend ER Discrimination	−0.03	0.04	0.51	0.02	0.04	0.67	−0.01	0.05	0.75
Friend ERI Dimension	0.07	0.04	0.07	0.15	0.04	0.00***	−0.02^e^	0.04	0.61
Sch Prom Cult Competence	0.09	0.05	0.08	0.08	0.05	0.13	0.09^f^	0.06	0.16
Sch Crit Cons Socialization	0.11^b^	0.06	0.07	0.09	0.05	0.08	0.00	0.06	1.00
Gender	0.05	0.03	0.16	0.01	0.04	0.70	−0.03	0.04	0.47
Generational Status	−0.09	0.04	0.03*	−0.09	0.04	0.02*	0.01	0.05	0.85
Grade 6 Multi-ethnic	0.10	0.06	0.09	−0.08	0.06	0.15	0.14	0.06	0.03*
Grade 9 Multi-ethnic	0.01	0.05	0.83	0.02	0.05	0.64	−0.08	0.05	0.13
White youth (*n* = 184)									
Own ER Discrim	0.08	0.04	0.02*	0.00	0.04	0.94	0.30^c^	0.09	0.00***
% Same-Group Friends	−0.05	0.04	0.27	−0.01	0.04	0.79	−0.11^d^	0.07	0.10
Friend Cultural Socialization	0.12^a^	0.07	0.09	0.13	0.04	0.00***	0.09	0.05	0.07
Friend ER Discrimination	−0.03	0.04	0.51	0.02	0.04	0.67	−0.01	0.04	0.75
Friend ERI Dimension	0.07	0.04	0.07	0.18	0.05	0.00***	−0.07^e^	0.07	0.30
Sch Prom Cult Competence	0.11	0.07	0.08	0.10	0.07	0.13	−0.13^f^	0.08	0.08
Sch Crit Cons Socialization	0.29^b^	0.09	0.00***	0.11	0.06	0.08	0.00	0.05	1.00
Gender	0.05	0.04	0.16	0.02	0.04	0.70	−0.02	0.03	0.47
Generational Status	−0.06	0.03	0.03*	−0.06	0.02	0.02*	0.00	0.02	0.85
Grade 6 Multi-ethnic	0.09	0.05	0.09	−0.08	0.05	0.15	0.09	0.04	0.04*
Grade 9 Multi-ethnic	0.01	0.05	0.83	0.03	0.06	0.64	−0.07	0.05	0.13

Parameters with the same superscript letter (e.g., a, b, c) represent significantly different coefficients between non-White and White youth based on chi-square difference tests for multigroup equality constraints. Friend ERI Dimension represent represents a sum composite of friend networks’ ERI Exploration as a predictor of focal youth ERI Exploration; similarly, a sum composite of friends’ ERI Resolution is a predictor focal youth ERI Resolution. Gender (1 = girl; 0 = boy). Generational status (1 = 1st generation, child and parents born abroad; 2 = 2nd generation, child born in the U.S. and two parents born abroad; 3 = 2.5 generation, one parent foreign-born and child and the other parent are U.S.-born; 4 = 3rd generation with U.S.-born child and parents). Standardized coefficients presented. Accounting for ethnoracial composition and developmental periods is done via 3 dummies: (1) Grade 6 Multiethnic (multiethnic middle school 1 and multiethnic middle school 2 combined) (2) Grade 9 Multiethnic (multiethnic high school 4), and (3) Grade 9 Majority White is used as a reference group

Note: ***p < 0.001, **p < 0.01, *p < 0.05

For ERI exploration, friend cultural socialization was significantly and positively associated with ERI exploration for ethnoracially minoritized youth (*ß* = 0.24, *p* < 0.001), and this path was nonsignificant for White youth (*ß* = 0.12, *p* = 0.09). In contrast, school critical consciousness socialization was significantly and positive associated with ERI exploration for White youth (*ß* = 0.29, *p* < 0.001), and this path was nonsignificant for ethnoracially minoritized youth (*ß* = 0.11, *p* = 0.07). These associations were documented above and beyond contributions of personal experiences of ethnoracial discrimination. Specifically, for both ethnoracially minoritized and White youth, own experiences of discrimination were significantly and positively associated with ERI exploration (*ß* = 0.08, *p* = 0.03). Considering other control variables, generational status was inversely related with ERI exploration such that youth with an increasing number of generations in the U.S. reported lower ERI exploration levels (*ß* = −0.09, *p* = 0.03 for minorized youth; *ß* = −0.06, *p* = 0.03 for White youth).

For ERI resolution, results indicated that there were no significant differences in friend and school socialization paths predicting ERI resolution between ethnoracially minoritized and White youth. For both ethnoracially minoritized and White youth, friend cultural socialization (*ß* = 0.12, *p* < 0.001 for minorized youth; *ß* = 0.13, *p* < .001 for White youth) and friend networks’ total levels of ERI resolution were significantly and positively associated with ERI resolution (*ß* = 0.15, *p* < 0.001 for minorized youth; *ß* = 0.18, *p* < .001 for White youth). With respect to control variables, results suggested that ERI resolution levels declined with an increasing generational status (*ß* = −0.09, *p* = 0.02 for minorized youth; *ß* = −0.06, *p* = 0.03 for White youth).

Finally, none of the hypothesized associations between friend and school factors were supported for ERI negative affect. Findings showed that the only significant associations that emerged in predicting ERI negative affect entailed two control variables. First, one path significantly differed between ethnoracially minoritized and White youth such that frequent personal experiences of ethnoracial discrimination were significantly and positively associated with ERI negative affect for ethnoracially minoritized youth (*ß* = 0.12, *p* = 0.02), and this path was also positive, but the magnitude of association was larger for White youth (*ß* = 0.30, *p* < .001). Second, 6th grade students attending multiethnic schools were more likely to report higher levels of ERI negative affect compared to 9^th^ grade students attending White majority school (*ß* = 0.14, *p* = 0.04 for minorized youth; *ß* = 0.09, *p* = 0.04 for White youth).

## Discussion

The growing ethnoracial diversity of the U.S. youth population (e.g., U.S. Census Bureau, 2021) underscores the importance of fostering ERI development because of its promotive and protective consequences for youth adjustment (Umaña-Taylor & Rivas-Drake, 2021). Informed by cultural-ecological models of minoritized youth development (García Coll et al., 1996; Hughes et al., 2016; Spencer, 2006) and the lifespan model of ERI (Williams et al., 2020), this study examined how friend and school ethnoracial socialization was associated with ERI development, and whether developmental period and ethnoracial minoritized/majority status moderated these associations. The current findings add to the literature by documenting similarities and differences in friend and school predictors of ERI exploration, resolution, and negative affect between ethnoracially minoritized and White youth. Specifically, friend cultural socialization was positively associated with ERI exploration only for ethnoracially minoritized youth, whereas school critical consciousness socialization was positively linked with ERI exploration only for White youth. Findings also indicated some similarities in the associations among friend ethnoracial socialization with ERI resolution for both groups. Specifically, friend cultural socialization and friend network’s levels of ERI resolution were positively associated with ERI resolution across ethnoracial groups and developmental periods (i.e., early versus middle adolescence). These friend and school socialization associations were documented above and beyond significant contributions of personal experiences of ethnoracial discrimination to ERI exploration and negative affect for both ethnoracially minoritized and White youth, which has tended to be the focus of past work (Brittian et al., 2015; Pahl & Way, 2006). Finally, these associations were tested while accounting for significant heterogeneity in ERI developmental processes by gender (Umaña-Taylor & Guimond, 2010) and immigrant generational status (Umaña-Taylor et al., 2013). With respect to these socio-demographic controls, gender was unrelated to ERI dimensions, whereas youth with an increasing number of generations in the U.S. reported lower ERI exploration and resolution levels, which replicates prior research. These findings, although awaiting replication with a more rigorous design, show that friend and school ethnoracial socialization processes uniquely inform ERI development and underscore the need to examine how socio-contextual dynamics shape ERI development across ethnoracially minoritized and White youth (Syed et al., 2018; Rogers et al., 2020; Williams et al., 2020).

### Contributions of Friend Network Characteristics to ERI Exploration and Resolution

Due to the proximal and salient nature of friends in adolescence (Brown & Larson, 2009) and their role in cultural and ethno-racial socialization (García Coll et al., 1996; Hughes et al., 2016; Spencer, 2006), it was anticipated that friend’s cultural socialization and friends’ ERI dimensions would each be positively associated with adolescents’ respective ERI dimensions. Further, friends’ experiences with ethnoracial discrimination (i.e., vicarious discrimination) were expected to be positively associated with ERI exploration and resolution. Finally, this study explored whether being embedded in friendship networks composed of a higher proportion of same-group friends with respect to ethnoracial background was linked to ERI developmental outcomes. The present findings provide evidence in partial support of these hypotheses and document some similarities and differences in the associations between friend socialization processes and ERI development for ethnoracially-minoritized and White youth.

Considering ERI exploration, the promotive effect of friend cultural socialization (i.e., discussing events and figures and reading books about one’s ethnic group) was detected only for ethnoracially minoritized youth, and not White youth. This suggests that for ethnoracial minoritized youth the extent to which they had explored their ERI was positively informed by their friends’ higher levels of engagement in activities to learn about the meaning of their ethnoracial group for their self-concept. This finding aligns with developmental models of ERI (e.g., Williams et al., 2020) and replicates past evidence from focus groups with ethnoracially minoritized youth for whom ERI exploration entails behavioral, affective, and cognitive engagement in learning though cultural events and activities, which is done in the company of friends (Sladek et al., 2022). This finding extends past research by documenting that youth are agentic in learning about their heritage culture in the company of their peers and friends, who play a vital role in transmitting cultural knowledge (Wang & Lin, 2023).

Interestingly, the association between friend cultural socialization and ERI exploration was nonsignificant for White youth in the present sample. Although the bivariate association between these constructs was significant and positive, when simultaneously considered with multiple friend and school predictors of ERI dimensions, it appeared that other factors (i.e., school critical consciousness socialization) were more important in catalyzing ERI exploration for White youth. Little is known about ERI development among White youth (e.g., Satterthwaite-Freiman & Umaña-Taylor, 2023) and the role of friends in ethnoracial socialization of White youth. Future work is needed to measure and identify the precise nature of socialization behaviors and messages exchanged among White friends because they are expected to differ from ethnoracial minoritized youth in their meaning-making of their ERI (e.g., focus on heritage or national identities rather than race). Such differences likely emerge due to society’s emphasis on white normativity perpetuating a perspective that White youth’s experiences are normative and require no interrogation and exploration (Moffit & Rogers, 2022).

Considering ERI resolution, results documented that having friends with whom youth frequently engaged in cultural socialization activities was consistently associated with higher levels of ERI resolution for all youth in the sample. These results lend quantitative support for prior qualitative findings from adolescent focus groups (Sladek et al., 2022). Taken together, these patterns underscore a potent role of friends in promoting heritage culture socialization, leading to increased behavioral and social engagement in learning about the meaning of one’s group membership (ERI exploration) as well as to increased clarity in the personal meaning of what one’s ethnoracial group membership means for one’s sense of self (ERI resolution). Although the documented association is consistent with insights regarding friend socialization and enactment of one’s ERI (Moffit & Syed, 2021; Sladek et al., 2022), the current findings are unique in their ability to link friend heritage cultural socialization mechanisms to adolescent ERI exploration and resolution, while accounting for multiple friendship and school factors as well as personal experiences of ethnoracial discrimination.

As expected, results showed a positive association between friend network’s ERI resolution and focal adolescent’s levels of ERI resolution. This association was consistent between ethnoracially minoritized and White youth and suggests that as adolescents were increasingly gaining clarity about the personal meaning of their ethnoracial group membership for their sense of self, they were also more likely to have friends who were reporting higher levels of ERI resolution. Whereas identity is a psychological construct that taps into an internal and private construction of the self-concept (Umaña-Taylor et al., 2014), ERI resolution may be communicated to friends via increased sense of confidence, agency, and comfort (Umaña-Taylor, 2016), which may explain the observed positive associations between higher levels of ERI resolution among friends and that of the focal individual. Although the present study did not examine social network dynamics of peer selection or influence, this pattern of positive associations among friend networks’ ERI resolution resonates with prior evidence from social network-informed studies of ERI development (Rivas-Drake et al., 2017).

In contrast, findings did not provide evidence that friend cultural socialization and friend network levels of ERI negative affect were associated with focal individual’s ERI negative affect. Given that this content dimension of ERI is relational and comparative in nature (Umaña-Taylor, 2024), it was hypothesized that messages adolescents receive from their salient and valued friends about how friends feel toward their own ethnoracial group could inform the degree to which focal youth have negative feelings about their own ERI. The present findings provide no support to these suppositions. Considering friend effects, it is possible that the cross-sectional nature of the data precluded this study from identifying the linkages between friends’ negative ERI and that of the focal adolescent because of its inability to test reciprocal associations between ERI and friend dynamics (i.e., ERI negative affect is associated with friend selection and friends influence each other’s ERI negative affect). Theoretically, negative ERI is expected to adversely impact self-concept, which could lead to a profile of psychological vulnerability and pose challenges in interpersonal relationships (Umaña-Taylor, 2024). This means that youth with higher levels of ERI negative affect could be less secure and confident, which can have an adverse impact on their ability to create and sustain their friendships (i.e., friend selection process). Future work needs to tease out the impact of ERI negative affect on friend networks selection and influence processes using longitudinal research designs.

Finally, given competing theoretical perspectives (Syed et al., 2018) and a mixed pattern of evidence on the role of friends ethnoracial composition in ERI development, the present study explored how proportion of same-ethnoracial group friends were related to any of the three ERI dimensions. Results indicated that the proportion of same-group friends was unrelated to ERI exploration, resolution, and negative affect. This lack of associations is not entirely surprising given that the present study examined more proximal and salient friend characteristics (e.g., friends’ socialization, friends’ ERI), which may be more impactful than structural features such as proportion of same-group friends that may represent a relatively more superficial aspect of friendship network context. In contrast to structural features captured by proportion of same-group friends, examining friend socialization and ERI captures a heterogeneous array of behaviors, messages, socialization inputs, and developmental processes that are exchanged among friends, in general, and same-group friends. As noted in the intergroup contact literature, though contact is a necessary feature, what is more significant for changing attitudes is the content of those peer interactions and relationships (Yip et al., 2019a). Applied to the current findings, having same-group friends may be important for informing ERI but it is the nature of engagement with those friends that ultimately may have the most impact (Wang & Lin, 2023). Thus, increasing specificity when conceptualizing and measuring *how* peers and friends of same- and different-ethnoracial groups shape adolescent ERI development is needed in future work.

### Contributions of School Socialization to ERI Exploration

Findings indicated that school critical consciousness socialization was promotive of ERI exploration among White, but not ethnoracially minoritized youth. This means that when White youth are provided with opportunities to engage with diverse peers and learn from school resources (e.g., events, educational sources) about group differences in power, privilege, and systems of oppression, they are more likely to be engaged in exploring and learning about the meaning of their ERI for their self-concept. These findings replicate and extend previous evidence documented among minoritized and White youth (Byrd, 2016). Although critical consciousness has been examined as a protective factor for the psychological and academic adjustment of ethnoracially minoritized youth (Heberle et al., 2020), when it comes to ERI development, the present results suggest that it is also critical for White youth. Documenting the association of school critical consciousness socialization is especially relevant for White youth because they might otherwise have limited opportunities for critical consciousness socialization in their family and community settings because White families often rely on schools to provide their children with ethnic-racial socialization experiences (Loyd & Gaither, 2018; Hamm, 2001). Further, this finding provides further evidence of the benefits of attending ethnoracially diverse schools for White students’ ethnic identity development (Brown et al., 2010). It is noteworthy that the association between school critical consciousness socialization and ethnoracially minoritized youth’s ERI exploration was positive and significant at a bivariate level, and decreased in the magnitude in the multivariate models. Taken together, these results suggest a relative greater importance of school critical consciousness socialization for ERI exploration among White youth, whereas cultural socialization with friends might be a stronger predictor for ethnoracially minoritized youth, as it likely builds upon earlier ethnoracial socialization in the family (Umaña-Taylor & Hill, 2020). Future research needs to disentangle developmental trajectories and cascades of ethnoracial socialization across multiple agents (i.e., family, peers, schools).

The present results indicated that school promotion of cultural competence was unrelated to ERI exploration, resolution, and negative affect. This is somewhat contrary to theoretical propositions that as youth receive more messages about the importance of being aware of diverse cultures in school they are more likely to engage in activities to explore and gain clarity in the personal meaning of their own ethnoracial group membership (Williams et al., 2020). Although school promotion of cultural competence was positively associated with ERI exploration at the bivariate level, it became nonsignificant when other friend and school predictors were considered. Perhaps the current measure of school promotion of cultural competence used, which focused on learning about other cultures and developing intercultural competence, is capturing a construct that would be more consequential for adolescents’ intergroup contact attitudes than their ERI development (Byrd & Legette, 2022). Indeed, Byrd and Legette (2022) also reported null associations between school promotion of cultural competence and ERI exploration and resolution, but significant and positive associations between this construct and adolescents’ other group orientation. It is possible that school promotion of cultural competence could be less effective in promoting one’s ERI development if this approach does not also integrate a recognition of the structural constraints faced by ethnoracially marginalized youth or discussions of commonalities in power struggles across groups that could help youth make sense of their ERI (Byrd & Legette, 2022).

Finally, it is plausible that the promotion of cultural competence measure does not capture an unmeasured heterogeneity in its delivery by school staff and the varied experiences that youth have in school, which could moderate its impact on ERI development. For instance, the quality of the teacher-student relationship may moderate the degree to which teachers are effective in promoting cultural competence, as it has been shown for ethnic-racial socialization processes in other proximal relationships, such as parent-adolescent dyads (Hernández et al., 2014). Another potential moderator entails student’s perspectives on whether the examples of promotion of cultural competence in the classroom are meaningful and engaging to adolescents (e.g., “tied to specific holidays/”heritage months,” or reactive – in response to events in the school or broader ethnic-racial climate; Byrd & Hope, 2020), as well as whether they are integrated into the curriculum in a culturally sustaining manner. Future research needs to further probe these aspects of the teacher-student relationship and students’ daily experiences and perspectives on school promotion of cultural competence to better understand the nuances of the effectiveness of school ethnoracial socialization.

### Developmental Implications

Despite a well-established literature noting the developmental significance of ERI and the role of parents in ethnoracial socialization processes (e.g., Huguley et al., 2019; Umaña-Taylor et al., 2014; Umaña-Taylor & Hill, 2020), the role of friends and schools in ERI development has been less clear. The present work shows that youth draw on cultural, racial, and social justice-related messages and knowledge transmitted by their friends and, to a lesser extent, schools to inform their feelings about, exploration, and finding clarity in what it means to be a member of their ethnoracial group. This study extends past research by documenting that youth are agentic in learning about their heritage culture in the company of their peers and friends, who play a vital role in transmitting cultural knowledge (Wang & Lin, 2023). The present study also provides preliminary evidence suggesting a relatively greater impact of school critical consciousness socialization for ERI exploration among White relative to ethnoracial minoritized youth, whereas cultural socialization with friends might be a stronger predictor for ethnoracial minoritized relative to White youth perhaps because it builds upon earlier ethnoracial socialization in the family (Umaña-Taylor & Hill, 2020). Future research needs to better understand that impact of developmental timing and unique socialization agents (i.e., school, peers, social media) for ERI exploration among White and ethnoracial minoritized youth.

This study underscores the potential influence of school-based friends in ERI development by highlighting that it may not necessarily be *who the friends are* that matters, but *what youth are doing with their friends* (in terms of engaging in behaviors, conversations, learning about cultural traditions, and celebrations) that is associated with higher ERI exploration and resolution levels. As such, these findings resonate with youth-centered accounts emerging from focus groups that reveal the rich and multifaceted role of friends in cultural socialization (e.g., Golden et al., 2022; Sladek et al., 2022). Indeed, friends and peers are theorized to play multiple roles in ERI development, including establishing and reinforcing behavioral norms, providing support and resources to cope with and resist ethnoracial discrimination, and participating in cultural activities and events, among others (Hughes et al., 2011; Wang & Lin, 2023). To properly characterize these many roles and interpersonal mechanisms underlying peer ethnic-racial socialization, developmental research needs to move beyond thinking of peers and friends merely from the perspective of interpersonal and structural diversity (i.e., proportion of same/cross group friends or peers in school; Yip et al., 2019a) to focus on specific *relational provisions* through which friends and peers facilitate or interfere with ERI development (Kornienko & Rivas-Drake, 2022; Kornienko et al., 2022) and ethnic-racial socialization (Wang & Lin, 2023). These goals could be accomplished by using advanced methods of social network analysis (e.g., Kornienko, 2024) and ecological momentary sampling and daily diary studies (e.g., Wang, 2020) to capture the rich tapestry and dynamics of peer relational provisions in adolescent lives.

### Translational Implications

The present findings have translational implications for educators, mentors, and school staff who need to cultivate social contexts in which youth could benefit from friend ethnoracial socialization. These findings suggest that schools and educators could intentionally create space for youth to engage with their peers, friends, and teachers in conversations and activities to learn and share about their cultural heritage and traditions. Given the null associations between the structural characteristics of friend groups and ERI development, it is not simply the promotion of other- vs. same-race/ethnicity friends that are promotive of ERI development, but the support of culturally specific exploration, engagement, and experiences related to their cultural identities that youth have together with friends and peers. When designing such school-based programs, educators need to use evidence-based developmental practices to ensure that these programs are not top-down, adult-led endeavors but rather are youth-centered to meet adolescent needs for autonomy, social status, and respect (Hoffman & Umaña-Taylor, 2023; Yeager et al., 2018). Finally, recognizing the importance of peer group norms and peer influence as promotive of ethnic-racial socialization is likely to be another component on which school staff need to be trained to counter widely accepted negative views of peer influence in youth development (Telzer et al., 2022).

Specific to youth of the ethnoracial majority, results indicated that school critical consciousness socialization was linked with higher ERI exploration for White youth; this suggests that interventions aimed at promoting critical consciousness socialization may be beneficial for ERI exploration among White students in multi-ethnic school settings. Furthermore, there was no evidence that this or other aspects of school cultural socialization were associated with ERI negative affect among White youth, which has been a concern in the current sociopolitical climate in which opponents of discussing race or ethnicity in the classroom, including critical race consciousness, argue that it promotes guilt and racial self-hatred among White youth (McGee, White, & Parker, 2021). As such, the current findings hold particular importance in the present sociohistorical context in the U.S. A robust body of evidence has shown that ERI development is a promotive and protective asset for minoritized youth (for meta-analytic evidence, see Miller-Coto & Byrnes, 2016; Yip et al., 2019b). Furthermore, these are vital developmental processes that occur within schools and support adolescent developmental needs for ethnic-racial identity development, relatedness, authentic school connection, academic success, future readiness, and health and wellbeing. Yet, even in settings in which schools and communities, including youth, are supportive of school-based opportunities to support students’ ERI development (e.g., Cabrera et al., 2013), state and federal policies can prohibit these efforts (Umaña-Taylor, *in press*). Thus, it will be critical to identify strategies to support educators, community, and youth who see the value of engaging in this work and want to embed it in school practices.

### Limitations and Future Directions

This study had a few limitations that represent directions for future research. First, participants only nominated friends who were part of their grade, so the results are generalizable only to school-based friend socialization of ERI development (except for frequency of cultural socialization that was measured for all peers). This network boundary prevented the study from incorporating important friendships that adolescents may have across grades or outside of school (Neal, 2020). Nonetheless, school-based friendships provide an opportunity to examine a peer context that is salient for youth, given the substantial amount of time that they spend together (Brown & Larson, 2009). It is important to underscore the scope of generalizability of the present results that are tied to a specific social, regional, and temporal context (i.e., predominantly Latinx public middle and high schools from the southwestern U.S. in 2019–2020, prior to COVID-19 pandemic). Future research needs to replicate the present findings and explore whether the strength of friend and school ERI socialization processes may vary for youth who are embedded in different contexts. In other words, sampling schools and geographic locales with differing ethnic-racial compositions compared to this study is needed to advance the knowledge of ERI socialization processes for youth from other ethnoracial backgrounds than predominantly Latinx context in the Southwest U.S. considered in this study. Finally, due to the cross-sectional nature of this study, the present study was unable to examine reciprocal and transactional associations between friend and school socialization and ERI development over time and future studies using longitudinal panel designs will need to replicate and elaborate on the present findings.

Future research will benefit from a stronger focus on interpersonal mechanisms unfolding among peers and friends that inform ERI development, including explicit (e.g., peer pressure, reinforcement, antagonistic teasing) and implicit (e.g., display of desired behaviors and creating opportunities for engagement with culture) peer socialization mechanisms (Hughes et al., 2011). In addition to these *social learning mechanisms*, more concerted efforts need to test *identity-based mechanisms* of how peers shape ERI development that entail emulation of valued others and adherence to social norms of a valued reference group (Brechwald & Prinstein, 2011). Because peer feedback and acceptance provide the foundation for a sense of self and identity development (Brechwald & Prinstein, 2011), the noted social learning and identity-based mechanisms can differentially impact exploring, resolving, and having negative affect about the meaning of one’s ethnic and racial group membership. For example, peer pressure and ethnoracial teasing may thwart ERI exploration and lead to ERI negative affect, whereas being embedded in a peer context that encourages engagement in cultural activities can catalyze ERI exploration and lead to ERI positive affect.

Future studies should more thoroughly attend to the distinct nature of school and peer socialization processes. Unlike socialization from peers and friends, where youth have more of a choice in whom they befriend and who they interact with, youth have relatively less choice in the schools they attend, where there is less opportunity for youth to select their teachers and school staff (e.g., counselors). Although friendships can be relatively more voluntary and egalitarian, relationships with educators are imbued with power differentials (Laursen & Bukowski, 1997), which may have implications for the strength and direction of ERI socialization effects. Clarifying these interpersonal mechanisms is necessary to advance developmental theory and inform interventions in ERI development.

## Conclusion

Building upon cultural-ecological and lifespan ERI development models, this study expands current understanding of how friend and school socialization mechanisms contribute to adolescent ERI exploration, resolution, and negative affect. This study reveals unique roles that friends and schools play in ethnic-racial socialization of adolescent’s exploration and sense of clarity about the meaning of race and ethnicity for their self-concept. The documented patterns highlight the need for developmental scholarship to move beyond the focus on who one’s friends are (i.e., compositional diversity) and toward a better understanding of *what interpersonal processes and social dynamics* unfold among youth and their friends and their consequences for ERI development. The present findings point to the key role that friend cultural socialization, friends’ ERI, and schools’ provision of learning opportunities to understand societal inequalities may play in promoting ERI development. Accumulating such evidence is vital to advancing developmental theory and informing interventions to foster ERI development.

## Footnotes

1 The term *ERI positive affect* is used to denote any measure that captures positive ERI affect, including ERI private regard and ERI affirmation to clearly distinguish from *ERI negative affect* (Umaña-Taylor, 2024).

2 Per APA recommendations, lowercase white is used when referring to a construct (e.g., whiteness). White is capitalized when in reference to people (e.g., White adolescents).
